# Early Oxidation Detection in White Wine by Electronic Tongue: A Preliminary Study

**DOI:** 10.1002/fsn3.70366

**Published:** 2025-05-27

**Authors:** Rachel I. Potter, Jungmin Lee, Carolyn F. Ross

**Affiliations:** ^1^ School of Food Science Washington State University Pullman Washington USA; ^2^ United States Department of Agriculture (USDA)‐Agricultural Research Service (ARS), Horticultural Crops Production and Genetic Improvement Research Unit Corvallis Oregon USA

**Keywords:** chardonnay, E‐tongue, rate‐all‐that‐apply, sensory, storage

## Abstract

In white wines, early detection of oxidation would alert winemakers to monitor potentially troubled wine more closely and take preventative measures to mitigate undesirable browning, flavors, and odors in their products. Current early oxidation detection methods include assessment by browning index, trained sensory panels, and quantification of byproducts such as quinones. The objective of this study was to assess the capability of the e‐tongue, a fairly new instrument that has previously been used to detect wine faults caused by spoilage organisms, in detecting early oxidative changes in Chardonnay wine. Clear bottles of Chardonnay were stored partially opened (treatment) in the dark at 2.2°C for 24 weeks. Wines were assessed at seven time intervals (0, 1, 2, 4, 8, 16, and 24 weeks) using the e‐tongue and a semi‐trained sensory panel with rate‐all‐that‐apply descriptors. Beginning at week 8 of storage, the e‐tongue discrimination indices (DI) between control and treated wine (sealed wine stored alongside partially opened wine bottles) were high (DI > 80%) and remained high throughout the study, indicating that the e‐tongue distinguished between control and treated samples. However, sensory panelists detected an increase in the intensity of vinegar/nail polish remover aroma attributes, attributes associated with wine oxidation, after 16 weeks of storage. These results suggest that the e‐tongue is a useful tool in the early detection of oxidized wine samples as compared to a sensory panel that perceived differences between control and treated wines 8 weeks after differences were detected by the e‐tongue.

## Graphical abstract

Beginning at week 8 of storage, the e‐tongue discrimination indices (DI) between control and treated wine (sealed wine stored alongside partially opened wine bottles) were high (DI > 80%) and remained high throughout the study, indicating that the e‐tongue distinguished between control and treated samples. However, sensory panelists detected an increase in the intensity of vinegar/nail polish remover aroma attributes, attributes associated with wine oxidation, after 16 weeks of storage. Alongside sensory testing, the e‐tongue shows promise for detecting early signs of oxidation.

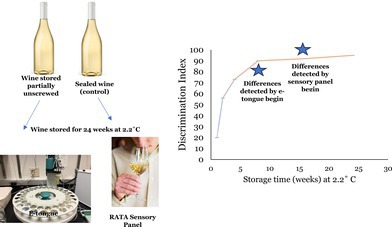

## 1 Introduction

Wine exposed to air can lead to the development of oxidative aroma and flavor notes in a wine (Ballester et al. 2018; Cejudo‐Bastante et al. 2011; Day et al. 2019; Pinto et al. 2018). In addition, oxidation causes color changes in wines, leading to less vibrant pigment in red wines or browning in white wines (Cartagena et al. 1994; Kramling and Singleton 1969; Voltea et al. 2022), all of which can have a negative impact on sensory perception. Sensory properties in an oxidized white wine can include loss of fruity aroma and an increase in unpleasant aromas including honey, solvent, dried fruit, banana, vegetal, rancid, and green apple (Ballester et al. 2018; Cejudo‐Bastante et al. 2011; Chisholm et al. 1995). These aromas can have a negative impact on a wine's perceived quality. Thus, it is important for winemakers to have reliable methods to detect the formation of these undesirable aromas to take corrective actions, if needed, before they become problematic.

Chemical progression of oxidation in wines can be assessed using several methods. Methods include monitoring volatile and non‐volatile compounds, while other methods follow color changes in the wine (Cheynier et al. 1991; Ferreira et al. 1997; Pinto et al. 2018). However, each of these methods focuses on specific components associated with oxidation, and individually, these methods fail to provide a holistic approach to monitoring wine quality. Advances in sensor technology have recently presented the use of the electronic tongue (e‐tongue) as a potential method to track wine faults (Paup et al. 2021; Diako et al. 2017).

The e‐tongue utilizes seven cross‐selective polycarbonate coated sensors that select for different ions or non‐volatile compounds that are associated with the five basic tastes (Nery and Kubota 2016). The e‐tongue potentiometric sensors display low selectivity and high cross sensitivity with high stability, making them an appropriate method for pattern recognition (Vlasov et al. 2005), with the potential to provide a holistic approach as a method for the detection of wine oxidation. Recent research has assessed the use of the e‐tongue for wine quality changes by spoilage organisms in red and white wines (Paup et al. 2021; Diako et al. 2017; Diako et al. 2016; Potter et al. 2024). The e‐tongue detected microbial wine faults in Merlot wine prior to flashprofiling by sensory panelists (Paup et al. 2021). The e‐tongue also distinguished Merlot wines with sub‐human detection threshold concentrations of 4‐ethylcatechol, a volatile compound indicative of *Brettanomyces* spoilage (Diako et al. 2017). Another study found that Riesling wine faults formed by spoilage microorganisms, including *Acetobacter aceti* , *Pediococcus parvulus* , *Wickerhamomyces anomalus*, and *Lactobacillus brevis* , were detected 1 week earlier by the e‐tongue when compared to a sensory panel (Potter et al. 2024). Extending this technology to additional white wine fault detection was the objective of this study, specifically to assess the ability of the e‐tongue to track early oxidative changes in white wines and to compare those data to those collected from a semi‐trained rate‐all‐that‐apply (RATA) sensory panel. Given previous findings (Paup et al. 2021; Diako et al. 2017; Diako et al. 2016; Potter et al. 2024), we hypothesized that while sensory panelists would detect differences in the white wine oxidation over storage, the e‐tongue would detect differences earlier.

## 2 Materials and Methods

### 2.1 Material Used and Their Sources

Sodium chloride, hydrochloric acid, and sodium‐L‐glutamate solutions were obtained from Alpha Mos (Tolouse, France) for e‐tongue conditioning, calibration, and diagnostics. Carlo Rossi Rhine white wine (Modesto, CA, USA) was obtained for e‐tongue calibration purposes. Chardonnay and Wine Faults kits were obtained for sensory panel training from Wine Awakenings (Niagara Falls, Ontario, Canada).

#### 2.1.1 Research Wines Used in Study

Clear, Burgundy‐shaped bottles of Columbia Valley barrel‐aged Chardonnay ( *Vitis vinifera* L.) from the 2021 harvest year were donated by Columbia Crest Winery (Paterson, WA, USA). Chardonnay was selected as the varietal used in this white wine study due to its economic prevalence in Washington State. According to the Washington Wine Commission (Washington State Wine Commission 2024), Chardonnay (including both barrel aged and non‐barrel aged) was the most popular white wine purchased in the state of Washington in 2022.

### 2.2 Wine Treatment and Storage

Clear bottles (*n* = 24) of Chardonnay with a screw top closure were stored halfway opened (treatment) to minimally introduce air exposure and oxidize wines. An estimation of partially opening the wine bottles to the “halfway point” was made by taking the screwcaps half the number of turns (1.5 turns) it took to fully seal the bottle (3 full turns). Control wines were defined as sealed bottles of Chardonnay wine that remained sealed until the first sampling point (*t* = 0 weeks, time 0). Partially opened bottles (sealed bottles opened at a time zero sampling point) were stored upright for 6 months in the dark at ~2.2°C, and evaluated at seven time points. At time 0, two bottles of wine were removed, opened, and analyzed as a control that day using a sensory panel and the e‐tongue. At the following analysis points (1, 2, 4, 8, 16, and 24 weeks), two bottles each of treated wines were randomly selected from storage.

### 2.3 Trained Sensory Evaluation Panel

The study protocol received approval from the WSU (Washington State University) Institutional Review Board (IRB) for conducting research with human subjects under IRB #19148–001.

#### 2.3.1 Sensory Panelist Training Prior to Sample Evaluation

Sensory panelists (*n* = 11, 8 women and 3 men, aged 22– 78, mean age = 34.9) participated in a trained RATA panel to assess oxidative changes in the wines over time. Panelists were recruited from a listserv of individuals who have previously participated in sensory panels at WSU. Panelists attended two 1‐h training sessions. Panelists were trained using aroma terms that were associated with oxidative spoilage, as well as known terms in unoxidized Chardonnay wine. At the first training session, aroma attributes including: apple/pear, honey, baking spices, vegetal, peach, vinegar/nail polish remover, banana, and butter were introduced to panelists. Panelists were also introduced to reference standards for these aroma attributes (Table 1) which were assessed using the RATA scale. An introduction to the RATA sensory ballot was conducted on Compusense (Guelph, Ontario, Canada) software, while panelists completed practice assessments of wine samples. At the first training session, as a group, panelists discussed and agreed on intensities for each of the aroma reference standards using the 3‐point RATA intensity scale (1 = low, 2 = medium, and 3 = high). It was clarified to panelists for aroma terms apple/pear and vinegar/nail polish remover that if only one component of either attribute was present, they should select it and rate the intensity of that one attribute (e.g., just pear or vinegar). Panelists were provided with a list of the aroma attributes described in Table 1 and were asked to take note of the intensity ratings agreed upon for each of the standards. Panelists were tasked with evaluating a replicate sample during the first training session. During the second training session, panelists reviewed each of the aroma standards prior to sample evaluations. After assessing each sample at the second training session, panelists discussed and came to a consensus on the aroma intensities.

**TABLE 1:** Aroma attributes and reference standards used in RATA (rate‐all‐that‐apply) training for sensory panelists who evaluated wine samples.

Aroma attribute	Standard composition
Apple/pear	5 g bartlett pear +5 g granny smith apple +5 g red delicious apple in 30 mL base wine^a^, ^b^
Vegetal (low)	5 mL canned green bean juice in 30 mL base wine^b^
Vegetal (high)	42 mL canned green bean juice in 30 mL base wine
Honey	6 g honey in 30 mL base wine^b^
Baking spices	0.1 g ground cinnamon and 0.1 g ground nutmeg in 30 mL base wine^b^
Butter	~0.05 g butter standard from Wine Awakenings Chardonnay kit^c^, in 30 mL base wine
Vinegar/nail polish remover	1 g drops of vinegar standard and ~1 g drops nail polish remover standard from wine faults Wine Awakenings kit^d^, in 30 mL base wine
Banana	0.05 g 99.9% drop isoamyl acetate in 30 mL of base wine

^a^ Base white wine, Carlo Rossi Rhine.

^b^ Standard composition created as followed by Willwerth et al. (2015).

^c^ Wine Awakenings Chardonnay kit, Niagara Falls, Ontario, Canada.

^d^ Wine Awakenings Wine Faults kit, Niagara Falls, Ontario, Canada.

Initial training was completed prior to sampling points at 0, 1, 2, 4, and 8 weeks. Refresher training occurred prior to both sampling points at 16 and 24 weeks. Panel composition changed due to scheduling conflicts at 16 weeks, but training remained consistent across all panelists.

Panelist performance was assessed by presenting panelists with replicates of a sample at each session. Two‐way analysis of variance (ANOVA) was used to assess consistency in aroma intensity ratings among replicates at each session. Feedback was provided to the panelists in the form of panel means, as well as specific feedback about their own performance. Panelists were asked to examine their attribute intensities in relation to the panel mean. Panelists with intensity means ±2 SDs from the panel mean were asked to practice with the aroma standard and re‐assess the sample. Feedback was provided to panelists after each training session.

#### 2.3.2 Trained Sensory Panel Analysis of Stored Samples

Sensory evaluation was completed under red lighting in partitioned booths at ambient temperature and pressure control at the WSU Sensory Science Center (Pullman, WA, USA). Trained panelists assessed treatment wines at six time points (1, 2, 4, 8, 16, and 24 weeks) and control wine bottles in replicate at time 0. Prior to each evaluation session, panelists reviewed aroma attribute standards presented during training.

At each time point, randomly selected Chardonnay treatment wines (*n* = 2 bottles) were poured 1 h prior to sensory evaluation in ISO glasses. At time 0, two sealed bottles (control) were opened 1 h prior to sensory analysis and served to panelists. The sensory analysis collected at time 0 was treated as a control. These glasses were capped with petri dishes and held at ambient temperature (~22°C) for 60 min before panel evaluation. Wine samples were presented with 3‐digit codes in a monadic sequential, randomized, and balanced order. Each sample was poured from a different bottle, resulting in panelists replicating bottle samples at each time point.

For each sample, panelists evaluated the presence of aroma attributes using terms: apple/pear, honey, baking spices, vegetal, peach, vinegar/nail polish remover, banana, and butter and the RATA scale (0 = absent, 1 = low, 2 = medium, and 3 = high). In addition, the RATA question listed an “other” aroma attribute option, in which panelists were then prompted to comment on the specific attribute that was noted. RATA attributes were presented on the ballot in a randomized order, except for the “other” option, which was always listed last. Panelists then rated the intensity of the selected aroma attributes. A 30 s break was provided between each sample to limit sensory fatigue.

### 2.4 E‐Tongue Analysis of the Wines

All Chardonnay samples were analyzed in triplicate using an e‐tongue (Astree II electronic tongue unit, Alpha MOS, Toulouse, France) within 1 h of sensory evaluation at each sampling point (*t* = 0, 1, 2, 4, 8, 16, and 24 weeks). Control wines (sealed and stored until e‐tongue measurements) were analyzed immediately after opening bottles sampled at time 0. The e‐tongue used in this study utilized seven cross‐selective sensors that were coated in a membrane and measured the potential difference between ions and molecules in a solution when compared to a reference electrode (Diako et al. 2017). This reference electrode contained an Ag/AgCl solution provided by the e‐tongue manufacturer (Alpha MOS 2011). Instrument preparation and sampling procedures were completed following manufacturer procedures and as previously described by Diako et al. (2016) and Paup et al. (2019). For e‐tongue analysis at each time point, three 25 mL beakers of replicated samples were analyzed (total *n* = 6 samples), and a reference/calibration sample (Carlo Rossi Rhine white wine) was included to account for instrument variability over time. Each set of wine measurements was separated with a 25 mL beaker of type 1 high‐quality water (18.2 MΩ‐cm) for a 10 s sensor cleaning. Six loops were completed during each run.

### 2.5 Statistical Analyses

XLSTAT (Addinsoft, Paris, France) software was used for sensory data analysis. STATA v. 18 (Stata corporation, College Station, TX, USA) was used to perform ordinary least squares regression on e‐tongue sensor signal data and aroma intensity data. Sensory data were analyzed by treating the RATA scale as continuous data and expanding the scale to 4 points (0 = absent, 1 = low, 2 = medium, and 3 = high) (Montero et al. 2020). A three‐way ANOVA was conducted to determine the effect of storage time, wine replicate (i.e., different bottle), and panelist on aroma attribute intensity. Mean aroma intensity values were separated using Fisher's Least Significant Difference (LSD) test with significance defined at *p* ≤ 0.05. To visualize sensory changes over time, Principal Components Analysis (PCA) was conducted. To account for the risk of type 1 error associated with running several ANOVA, a MANOVA was also conducted (Lawless and Heymann 2010). MANOVA was used to determine the effect of wine storage time, replicate, and panelist on aroma attribute intensities. Wilks' Lambda Test was run to assess the effect of storage time, wine replicate, and panelist on Chardonnay aroma attribute intensities.

E‐tongue data were analyzed using Astree Alphasoft software (ver. 12, Alpha MOS) to calculate the Discrimination Index (DI), a measure of overlap among samples and distance between samples, and to visually determine differences using PCA. A negative DI indicates overlap, and a positive DI indicates separation. A DI greater than or equal to 80 indicates that the e‐tongue could detect strong differences (Alpha MOS 2011). E‐tongue data were extracted from the fourth loop of each run. Each sample was represented on the PCA plot as a triangle of three data points to achieve relative standard deviation (RSD) < 15%. DIs were determined between stored Chardonnay wines at all seven time points to determine differences in the wines with respect to time. Chardonnay wine sampled at time 0 was treated as a control in PCA models.

To further understand relationships between e‐tongue results (DI) and intensity of RATA attributes, Pearson's correlation analysis was conducted. Additionally, ordinary least squares (OLS) regression was conducted to determine if significant relationships exist between e‐tongue sensor signals and aroma intensities. Significance was defined at *α* = 0.05.

## 3 Results

### 3.1 RATA Sensory Panel Findings

Storage time affected the aroma attributes in the stored Chardonnay wine. Specifically, the aroma attribute vinegar/nail polish remover increased significantly (*F* = 6.18, *p* ≤ 0.001) over the 24‐week storage period (Table 2). This increase in vinegar/nail polish remover aroma began after 16 weeks of storage at 2.2°C, scoring an intensity of 1.64, an intensity significantly higher than the intensity observed in unoxidized control wine sampled at time 0 (0.36). Vinegar/nail polish remover aroma remained significantly higher than what was observed at time 0 for the remainder of the study. The intensity of aroma attribute vinegar/nail polish remover was 1.82 after 24 weeks of storage, approximately equivalent to a medium intensity on the RATA scale (2 = medium).

**TABLE 2:** Aroma intensity responses to treated Chardonnay wines, stored partially opened at 2.2°C, during 24 weeks of storage as assessed by a semi‐trained rate‐all‐that‐apply (RATA) panel. The *p*‐value listed within a column represents the significance of storage time on the intensity of that aroma attribute as determined using analysis of variance (*α* = 0.05).

Storage time (weeks)	Apple/pear	Vegetal^a^	Honey	Peach	Baking spices	Vinegar/nail polish remover	Banana	Butter
0	0.54 abc ± 0.77	1.14 ± 1.21	0.43 a±0.84	0.57 ± 0.84	0.07 ± 0.26	0.36 bc ± 0.62	0.39 ab ± 0.83	0 ± 0
1	0.18 c ± 0.59	1.05 ± 1.13	0.18 ab ± 0.39	0.50 ± 0.74	0.05 ± 0.21	0.55 bc ± 0.80	0.73 a ± 0.94	0 ± 0
2	0.39 bc ± 0.80	1.08 ± 1.29	0.23 ab ± 0.51	0.39 ± 0.70	0 ± 0	0.73 b ± 0.92	0.42 ab ± 0.81	0 ± 0
4	0.32 bc ± 0.78	1.18 ± 1.14	0.36 ab ± 0.73	0.41 ± 0.85	0 ± 0	0.23 c ± 0.43	0.46 ab ± 0.91	0.09 ± 0.29
8	0.90 a ± 1.02	0.90 ± 1.07	0.50 a ± 0.69	0.60 ± 0.75	0.05 ± 0.22	0.45 bc ± 0.83	0.15 b ± 0.67	0.15 ± 0.37
16	0.68 ab ± 0.89	0.68 ± 0.95	0.32 ab ± 0.57	0.73 ± 0.83	0.05 ± 0.21	1.64 a ± 0.85	0.14 b ± 0.47	0.14 ± 0.35
24	0.59 abc ± 0.67	0.77 ± 0.87	0.09 b ± 0.29	0.50 ± 0.60	0 ± 0	1.82 a ± 1.01	0.14 b ± 0.47	0.14 ± 0.47
*p*	< 0.0001	< 0.0001	0.038	< 0.0001	0.057	< 0.0001	0.001	0.138

*Note:* A different letter within a column represents a significant difference within that aroma attribute as determined using Fisher's Least significant difference (*p* ≤ 0.05).

^a^ Attributes with no letters next to mean values had no significant differences across time as determined by Fisher's LSD.

Honey aroma intensity decreased over storage time (*F* = 1.69, *p* ≤ 0.05). Control wines sampled at time 0 had a honey aroma intensity of 0.43, and this intensity significantly decreased to 0.09 by week 24 of storage (Table 2).

**FIGURE 1:**
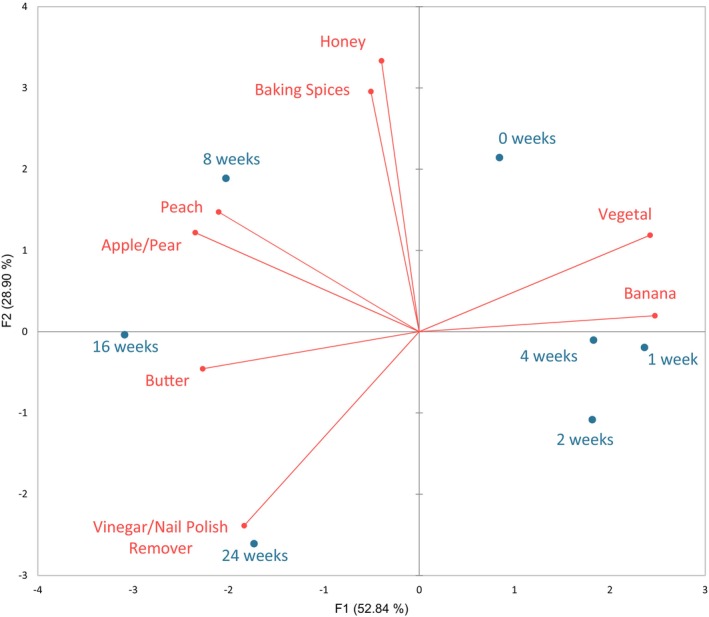
Principal components analysis of treated Chardonnay wines, bottles stored partially opened at 2.2°C and assessed at seven intervals during 24 weeks by a semi‐trained rate‐all‐that‐apply (RATA) panel. The storage time of treated wines is presented with blue labels. Red vectors and labels indicate RATA aroma attributes.

Visualized aroma changes in wine over storage time by PCA is shown in Figure 1. PC1 and PC2 explained 82.95% of the variation within the model. PC1, which explained 52.46% of the variation in the model, was positively loaded with the aroma attributes vegetal and banana, and negatively loaded with the aroma attribute butter. PC2, which explained 30.49% of the variation in the model, was positively loaded with aroma attributes of honey and baking spices. Wines after 1, 2, and 4 weeks of storage were characterized by the banana attribute. Wines after 8 weeks were characterized by apple/pear and peach aromas, while after 24 weeks they were characterized by the vinegar/nail polish remover attribute.

Significant positive relationships were noted between the variables across the 24‐week storage time (Table 3). The intensity of the aroma attribute vinegar/nail polish remover had a significant positive relationship (*r* = 0.90) with storage time (weeks). Additionally, the intensity of the aroma attribute banana had a significant negative relationship (*r* = −0.76) with storage time (weeks), indicating that as storage time progressed, banana aroma intensity decreased.

**TABLE 3:** Pearson correlation analysis between variables including aroma attribute intensities assessed by a rate‐all‐that‐apply panel, e‐tongue discrimination indices (DI), and storage duration (number of weeks) for treated Chardonnay wine during 24 weeks of storage.

Variables	Apple/pear	Vegetal	Honey	Peach	Baking spices	Vinegar/nail polish remover	Banana	Butter	DI
Apple/pear	**1.00**	−0.61	0.50	0.65	0.32	0.30	**−0.89**	0.72	0.54
Vegetal	−0.61	**1.00**	0.25	−0.68	−0.04	**−0.88**	0.73	−0.72	−0.68
Honey	0.50	0.25	**1.00**	0.35	0.56	−0.59	−0.19	0.14	−0.08
Peach	0.65	−0.68	0.35	**1.0**	0.70	0.40	−0.53	0.47	0.19
Baking spices	0.32	−0.04	0.56	0.70	**1.00**	−0.24	0.04	−0.15	−0.50
Vinegar/nail polish remover	0.30	**−0.88**	−0.59	0.40	−0.24	**1.00**	−0.60	0.50	0.56
Banana	**−0.89**	0.73	−0.19	−0.53	0.04	−0.60	**1.00**	**−0.82**	−0.74
Butter	0.72	−0.72	0.14	0.47	−0.15	0.50	**−0.82**	**1.00**	**0.89**
DI	0.54	−0.66	−0.08	0.19	−0.50	0.56	−0.74	**0.89**	**1.00**

*Note:* Values in bold denote a significant correlated relationship between two variables (*p* ≤ 0.05).

### 3.2 E‐Tongue Findings

**FIGURE 2:**
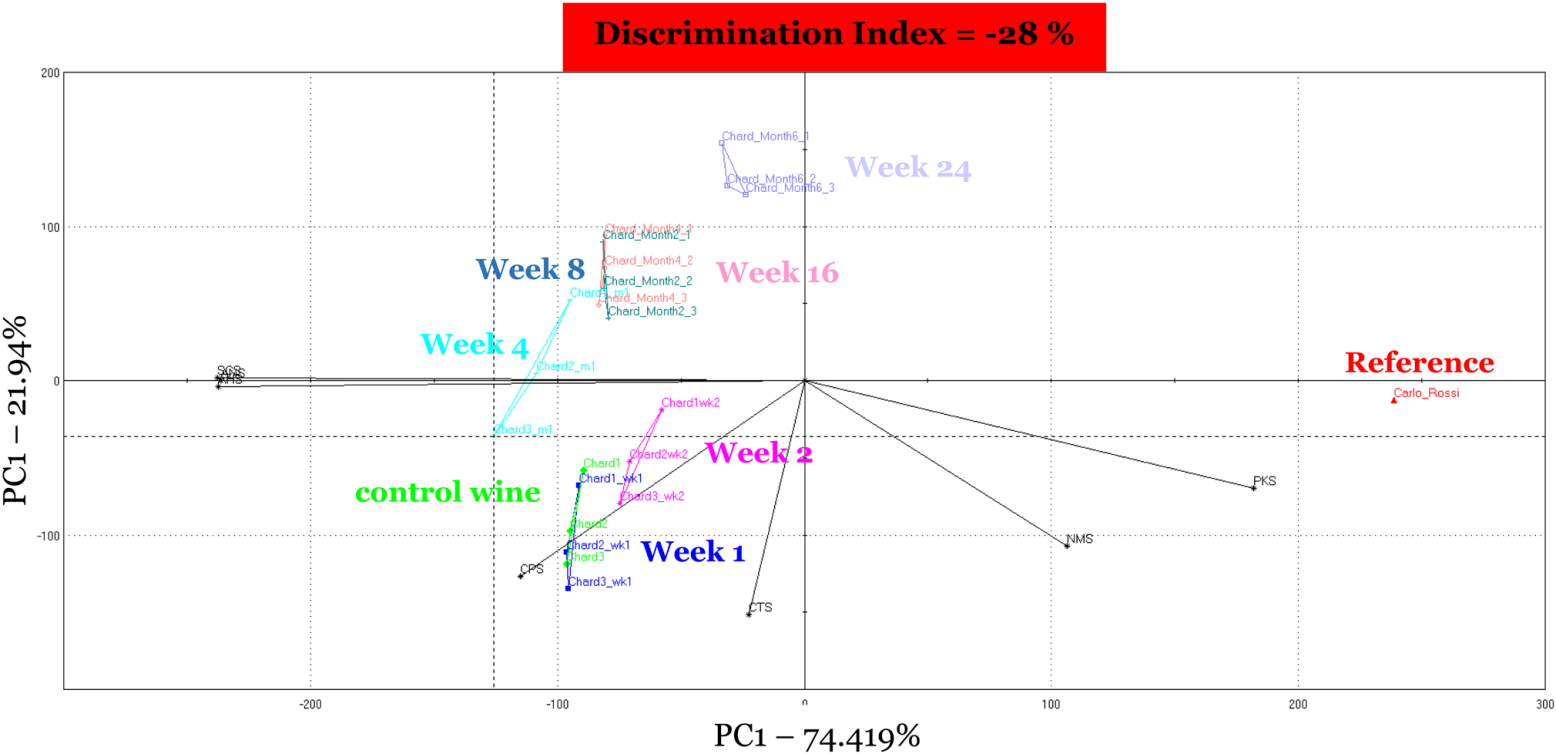
Principal components analysis of treated Chardonnay wines, stored under conditions to promote oxidation at 2.2°C for 24 weeks as assessed using the e‐tongue. Results at the seven storage time points are shown as *t* = 0, 1, 2, 4, 8, 16, and 24 weeks. Carlo Rossi Rhine wine was used as an instrument reference/calibration (in red) at each analysis timepoint to account for instrument variation. E‐tongue sensor vectors (in black) are indicated by sensors: AHS, PKS, CTS, NMS, CPS, ANS, and SCS. Control wine (in green) was a freshly opened bottle of the Chardonnay wine at each sampling point in this study.

**FIGURE 3:**
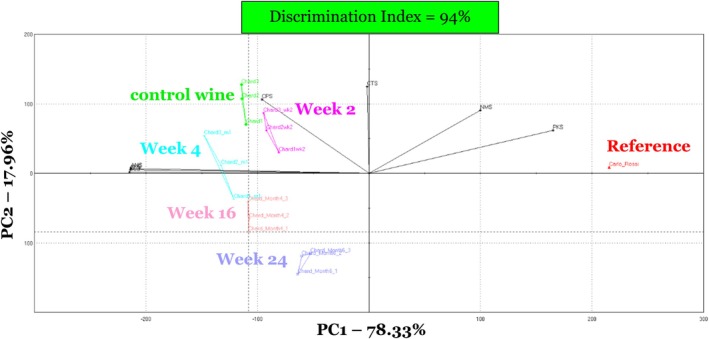
Principal components analysis of Chardonnay wine, stored under conditions to promote oxidation at 2.2°C for 24 weeks as assessed using the e‐tongue. Storage point weeks 1 and 8 removed in the PCA plot. Carlo Rossi Rhine wine was used as an instrument reference (in red) at each analysis point to account for instrument variation. E‐tongue sensor vectors (in black) are indicated by sensors: AHS, PKS, CTS, NMS, CPS, ANS, and SCS. Control wine (in green) was a freshly opened bottle of the Chardonnay wine at each sampling point in this study.

When all seven time points (*t* = 0, 1, 2, 4, 8, 16, and 24 weeks) were included in the PCA model, the e‐tongue had a DI of −28% (Figure 2). A negative DI indicated that sample areas on the PCA overlapped, suggesting that the samples were too similar in soluble compound composition. Overlap was observed between unopened (control) wine sampled at time 0 and treated wines after 1 week of storage. In addition, there was overlap between treated wines stored for 8 and 16 weeks. To further determine if good separation existed between stored wines on the PCA model with respect to time, samples that overlapped including weeks 1 and 8 were removed from the model. When treated wines at weeks 1 and 8 were removed from the PCA model, DI increased from −28% to 94%, indicating good separation (Figures 1 and 3).

To further understand the differences among the Chardonnay samples during storage, the Euclidian distances and DIs were evaluated at each time point (*t* = 0, 1, 2, 4, 8, 16, and 24 weeks) (Table 4). Wines sampled at storage weeks 1, 2, and 4 showed poor separation when compared to wines sampled at time 0, with DIs of 20%, 56%, and 73%, respectively. Wines sampled at weeks 8, 16, and 24 all showed good separation from wines sampled at time 0 (control), producing DIs of 90%, 92%, and 95%, respectively. These results indicate that the e‐tongue detected differences due to oxidation in wines beginning at 8 weeks of storage and remained significantly different throughout the duration of the study.

**TABLE 4:** Discrimination indices (%, DI) between wines displayed in Figure 2 produced by the e‐tongue.

Sample	Reference	Euclidean distance	DI
Week 1	Control	30.1	20
Week 1	Week 2	72.2	56
Week 1	Week 4	134.7	78
Week 1	Week 8	171.0	91
Week 1	Week 16	179.8	92
Week 1	Week 24	247.6	96
Control	Week 2	71.4	56
Control	Week 4	117.5	73
Control	Week 8	156.9	90
Control	Week 16	168.3	92
Control	Week 24	236.7	95
Week 2	Week 4	97.7	64
Week 2	Week 8	121.9	83
Week 2	Week 16	133.8	86
Week 2	Week 24	191.8	92
Week 4	Week 8	79.5	60
Week 4	Week 16	115.6	77
Week 4	Week 24	166.4	87
Week 8	Week 16	46.4	55
Week 8	Week 24	94.6	83
Week 16	Week 24	95.0	84

*Note:* Rows highlighted in gray represent samples that showed good separation (> 80% DI). Control represents a sealed bottle of Chardonnay wine stored alongside treated wines opened at each sampling point.

While conducting OLS regression, significant relationships were found between e‐tongue sensor signals and vinegar/nail polish remover, honey, and vegetal aroma intensities (*p* ≤ 0.05). All seven e‐tongue sensor signals were found to be significant in predicting vinegar/nail polish remover aroma in the stored wines (Table 5). Coefficients for vinegar/nail polish remover aroma and e‐tongue sensor signals were negative for all OLS regression models (Table 5). These negative coefficients indicate that as vinegar/nail polish remover aroma increased, the sensor signals for each of the seven e‐tongue sensors decreased. Additionally, all e‐tongue sensors were significant in predicting honey aroma intensity. All coefficients between e‐tongue sensory signals and honey aroma intensity were positive, indicating that as honey aroma increased, the e‐tongue sensor signals were also predicted to increase. Specific e‐tongue sensors, including sensors CTS, CPS, and ANS, were found to be significant in predicting vegetal aroma intensity. Regression models including CTS, CPS, or ANS sensors and vegetal aroma intensity all had positively correlated results, indicating that as vegetal aroma increased, the e‐tongue sensor signals were also predicted to increase.

**TABLE 5:** Ordinary least squares (OLS) regression correlation coefficients between e‐tongue sensor signals and aroma intensities as assessed by a RATA (rate‐all‐that‐apply) sensory panel for wines stored partially opened at 2.2°C for 24 weeks.

Variables	Sensor AHS	Sensor PKS	Sensor CTS	Sensor NMS	Sensor CPS	Sensor ANS	Sensor SCS
Apple/pear	7.29 e‐06	0.000043	−0.000019	0.000091	−0.000031	−0.000053	−4.94E‐06
Vegetal	0.00015	0.000017	**0.00016**	0.000053	**0.00020**	**0.00019**	0.00015
Honey	**0.00021**	**0.00015**	**0.00018**	**0.00018**	**0.00020**	**0.00018**	**0.00020**
Peach	−3.23E‐06	0.000057	0.000014	0.000027	−0.000025	−0.000018	−8.62E‐06
Baking spices	8.10E‐06	0.000018	0.000019	5.65E‐06	6.82E‐06	1.13E‐05	6.23E‐06
Banana	0.000091	0.000076	0.00012	−6.33E‐06	0.00012	0.00015	9.42E‐05
Butter	6.53E‐06	0.000039	−0.000021	0.000041	−7.88E‐06	−0.000027	5.37E‐06
Vinegar/nail polish remover	**−9.15E‐04**	**−0.00067**	**−0.00080**	**−0.00062**	**−0.00099**	**−0.00094**	**−8.88E‐04**

*Note:* Values in bold denote a significant relationship between two sensors and attributes (*p* ≤ 0.05).

## 4 Discussion

### 4.1 Sensory Panel Responses

These sensory findings were similar to those previously reported on oxidized white wines. The oxidation of ethanol to acetaldehyde and then to acetic acid in white wines is well understood in the wine industry (Bai et al. 2024; Coetzee, Van Wyngaard, et al. 2016; Gabrielli et al. 2021; Oliveira et al. 2011). Acetaldehyde, a precursor to acetic acid formation, has previously been described as having an aroma similar to freshly cut apples in white wines (Coetzee, Brand, et al. 2016). This may explain why these wines were characterized by having an apple/pear aroma after 8 weeks (Figure 1) before vinegar/nail polish remover aroma intensity increased significantly after 16 weeks. However, in order to confirm this, quantification would need to be conducted, and acetaldehyde was not quantified in the present study. Acetic acid, a compound with a vinegar aroma, is associated with oxidation (Baena‐Ruano et al. 2010; Rossetti and Boselli 2017). A previous accelerated oxidation study found that when bottles of white wine, including Riesling and Sultana wines that were stored at 50°C in the presence of air for 4 weeks, a significant increase in acetic acid was found (Simpson 2016). Thus, it was expected in the present study that the treated wines stored for a longer period (an additional 20 weeks compared to Simpson 2016), the intensity of vinegar/nail polish remover aroma The significant increase in vinegar/nail polish remover aroma that occurred is also supported by the results found while conducting OLS regression (Table 5). Vinegar/nail polish remover aroma intensity had significant relationships with all e‐tongue sensor signal intensities. An increase in volatile acidity is associated with oxygen exposure in white wines, including barrel‐aged Chardonnay and Debina varietals (Rossetti and Boselli 2017; Vaimakis and Roussis 1996); thus, these relationships suggest significant changes in our treated wines were also due to oxidation.

The development of wine faults including oxidation is also typically accompanied by a loss or reduction in expected attributes for a wine varietal (Grainger 2023). One previous study quantified esters, norisoprenoids, and terpenes in 48 different Chardonnay wine samples (Arrhenius et al. 1996). These data were then correlated with aroma attributes developed from a descriptive analysis sensory panel (Arrhenius et al. 1996). This study found that honey aroma, along with compounds that were significantly correlated with this aroma, were important chemical markers expected to be present in Chardonnay wines (Arrhenius et al. 1996). Given that honey is expected in the positive aroma profile for Chardonnay, a reduction in *honey* aroma, which occurred in the present study, may be viewed as a fault in oxidized Chardonnay wines. Another study found that volatile ester levels decreased with time in Chardonnay wines stored under oxidative conditions (Patrianakou and Roussis 2013). This study found that volatile esters, including isoamyl acetate concentration, decreased with respect to time in Chardonnay wines stored under conditions to promote oxidation. This aligns with what we observed in the present study, given that banana aroma had a significant negative relationship with storage time (*r* = −0.76).

### 4.2 E‐Tongue Responses

The e‐tongue responses were further explored to identify if a given sensor was useful in distinguishing the stored wines. The e‐tongue sensors are not exclusively selective for one particular compound in solution; rather, they respond to many different analytes (Vlasov et al. 2005). Previous work found that an “all‐solid‐state” e‐tongue could successfully monitor the early production of acetic acid in dry white wine varietals, including Monte Schiavo, Casal de'Cavalieri, Caldirola, Sartarelli, Fazi Battaglia, Piersanti, and Sant'Ignazi (Verrelli et al. 2007). Based on findings from Verrelli et al. (Verrelli et al. 2007), good separation between stored and control wines beginning on week 8 (Table 4) could have been from the start of acetic acid formation in the wine prior to when panelists detected significant increases in vinegar/nail polish remover aroma intensity on week 16 of storage; although, again, acetic acid quantification is necessary to confirm this.

Literature on wine fault detection by e‐tongue is limited, with minimal research on white wines available. Previous work has shown strong correlations between the e‐tongue response and sensory measurement. In red wine, the e‐tongue showed strong relationships (*r* ^2^ > 0.93) among the sensory perception of sweet, sour, bitter, burning, astringent, and metallic and the response by specific e‐tongue sensors (Diako et al. 2016). Additional work has also shown that the e‐tongue could detect differences at 3 weeks in Merlot wines inoculated with spoilage microorganisms prior to a flash profiling sensory panel, where differences were apparent at 4 weeks (Paup et al. 2021). Another study found that the e‐tongue detected sub‐threshold concentrations of 4‐ethylcatechol in Merlot wines when linked to the consumer detection threshold (Diako et al. 2017). Another study looking at red wine phenolic composition related their results to data collected from a trained sensory panel and found that the e‐tongue was good at predicting the overall quality of dry red wines (Rudnitskaya et al. 2010). An additional study was also conducted looking at the capabilities of an e‐tongue and electronic nose (e‐nose) in measuring oxygen levels and polyphenol content in red wine (Rodriguez‐Mendez et al. 2014). This study found that the e‐tongue and e‐nose showed good correlations with oxygen levels in red wine.

Additionally, there is a limited selection of literature available on the use of the e‐tongue for analyzing white wines (Potter et al. 2024; Gutiérrez et al. 2011). Previous work found that an e‐tongue was capable of distinguishing between white wine samples including Chardonnay by grape geographic origin (Gutiérrez et al. 2011). A storage study conducted that assessed the e‐tongue's capabilities in detecting early signs of microbial spoilage in Riesling wine found that the e‐tongue was capable of detecting differences 4 weeks prior to a RATA sensory panel (Potter et al. 2024). The significant relationships found in the present study between sensor signals and aroma intensities are supported by these prior studies that also found significant relationships between the e‐tongue and sensory measurements. Specifically, the significant relationships found between vinegar/nail polish remover aroma and e‐tongue sensor signals in the present study suggest that the e‐tongue may be a useful tool for detecting early changes due to oxidation in Chardonnay wines.

Previous research has shown the e‐tongue shows good correlation with sensory perceptions and has been more sensitive than sensory panels in the detection of early wine fault development and also in determining overall wine quality (Paup et al. 2021; Diako et al. 2017; Diako et al. 2016; Potter et al. 2024; Rudnitskaya et al. 2010). In the present study, the e‐tongue was able to detect differences in soluble compound composition beginning on week 8 of storage when compared to control wine sampled at time 0. However, trained sensory panelists were only able to detect differences in spoilage term vinegar/nail polish remover intensity beginning on week 16 of storage. It is important to analyze wines using the e‐tongue alongside sensory testing to further understand which sensory changes are happening and to provide the winemaker with an opportunity to further explore the chemical changes occurring within affected wines. These results along with previous findings suggest that the e‐tongue may be a useful tool for detecting early development of oxidation in white wines when analyzed alongside sensory testing.

### 4.3 Limitations of This Study and Future Directions

This study demonstrated that the e‐tongue could be used to distinguish white wine changes over storage time. However, this study had limitations relating to the sensory panel; due to the length of the 24‐week storage study, panel composition varied between time points 0–8 weeks to 16 and 24 weeks. It would have been ideal to have the same semi‐trained panelists participate at all time points across the 24‐week storage period to minimize the possible effect of panelist response variation on scoring aroma attribute intensity. Future studies could include recruiting a consumer sensory panel with a larger number of participants and repeating the current work or conducting a descriptive analysis panel with the same panelist composition at all time points. This study could also focus on collecting data between the points of analysis where sensory changes began to occur (*t* = 0, 8, 16, and 24 weeks) using the check‐all‐that‐apply (CATA) method, because RATA is not an appropriate method for a consumer sensory panel. In addition, wines have been analyzed by a consumer sensory panel in the weeks just before and after changes were detected (*t* = 8 and 16 weeks; Table 2) using the e‐tongue and a semi‐trained sensory panel in the current study (*t* = 7, 9, 15, and 17 weeks) to further understand the aromatic changes in the wines before changes were detected in the present study. This would provide further insight into the specific time points at which sensory changes begin to occur in the wines. Additionally, the Chardonnay used for this study was an oaked Chardonnay, which is a style of Chardonnay that has decreased in popularity over the last 20 years.

Future studies could include additional time points between 8 and 16 weeks of storage to determine more precisely when the panel could begin to detect sensory differences in treated wines. In addition, a wider range of temperatures could be tested in future studies to assess the effect of temperature on oxidative aroma intensity. Previous studies looked at the effect of temperature on browning in bottled white wine varietals, including Sauvignon Blanc, Chardonnay, Trebbiano, Albana, and Muller‐Thurgau, and found that the onset of browning followed an Arrhenius‐like dependence on temperature (Ricci et al. 2017). No work has compared color compound changes and e‐tongue potentiometric sensor responses. Future studies could modify storage conditions to better reflect consumer and processor handling of wines. Different bottle colors could also be considered.

This study was also limited in the analytical characterization of wine. Future work could include measuring the polyphenolic composition of the base wines prior to oxidation as previous work has shown that, since high concentration of 2‐*S*‐glutathionyl caftaric acid in Chardonnay wines has been shown to have links to increased intensity of oxidative notes (Ballester et al. 2018). Aroma compounds including furfural, acetic acid, ethyl acetate, isoamyl acetate, and/or hexanal have previously been associated with oxidative aromas in young white wines including Chardonnay wine (Escudero et al. 2002). These compounds could have been quantified prior to and after storage using Gas Chromatography–Mass Spectrometry to further understand the changes in specific aroma compounds with respect to time. In addition, titratable acidity, Brix, browning index, percent alcohol, and total dissolved oxygen could have been measured to better understand additional chemical changes in the wines with respect to time.

It must also be noted that the e‐tongue separates samples based on their non‐volatile profile, with limited research available on the e‐tongue's capabilities in discriminating volatile compounds (Diako et al. 2017). Over the 24‐week oxidation study, wines were only assessed for aroma by panelists. Given that the e‐tongue determines differences among samples based on soluble and insoluble compounds (Diako et al. 2017), this makes it difficult to directly compare RATA and e‐tongue results. Previous work has found that the e‐tongue was able to detect sub‐human threshold concentrations of 4‐ethylcatechol in Merlot wines when compared to the consumer detection threshold (Diako et al. 2017). Given that the e‐tongue has previously been capable of discriminating among red wine samples spiked with low concentrations of 4‐ethylcatechol, this implies that the e‐tongue may have the potential to detect changes that occurred due to volatile compound spiking. To determine if the e‐tongue could detect oxidation indicator compounds, wines could have been spiked with these compounds (e.g., furfural, acetic acid, acetaldehyde, ethyl acetate, isoamyl acetate, or hexanal) at concentrations below sensory thresholds and analyzed. Future studies can include further application of the e‐tongue to detect volatile compounds at various human sub‐threshold levels.

## 5 Conclusion

Significant sensory differences (*p* ≤ 0.05) occurred in Chardonnay wine stored at 2.2°C across the 24‐week storage period. Specifically, there was an increase in vinegar/nail polish remover aroma after wines were stored for 16 weeks, and honey aroma decreased after 24 weeks of storage. Additionally, the e‐tongue was also able to discriminate against Chardonnay wine stored at 2.2°C in wine bottles stored halfway open and sampled for 24 weeks. DIs between wines stored at 2.2°C and controls were high (DI > 80%) beginning on week 8 of storage and remained high for the entire duration of the study. This was 8 weeks prior to the time point at which significantly higher intensity of oxidative aroma terms, including vinegar/nail polish remover, were determined by a RATA panel (week 16). The preliminary findings of this study show that alongside sensory analysis, the e‐tongue has the potential to be a useful tool in the early detection of oxidation in white wines by detecting changes prior to a sensory panel; however, additional research is needed to further validate the e‐tongue's capabilities in oxidation detection. Future research studies can expand upon the present study by including packaging as an independent variable and also monitoring changes in color with respect to time.

## Author Contributions

**Rachel I. Potter:** data curation (equal), formal analysis (equal), investigation (equal), methodology (equal), visualization (equal), writing – original draft (equal), writing – review and editing (equal). **Jungmin Lee:** conceptualization; investigation; writing – original draft; methodology; writing – review and editing; formal analysis. **Carolyn F. Ross:** conceptualization; investigation; funding acquisition; writing – original draft; methodology; writing – review and editing; visualization; project administration; supervision; resources; data curation.

## Ethics Statement

This wine sensory panel was approved by the Institutional Review Board of WSU (IRB #19148‐001), with written informed consent obtained from all study participants.

## Conflicts of Interest

The authors declare no conflicts of interest.

## Supporting information


Data S1.

## Data Availability

The data that support the findings of this study are available from the corresponding author upon reasonable request.
