# Supplementary material for: Early Oxidation Detection in White Wine by Electronic Tongue: A Preliminary Study
Source: Food Sci Nutr. 2025 May 27;13(6):e70366. doi: 10.1002/fsn3.70366 (PMC12121522; doi:10.1002/fsn3.70366)
Supplement: Supplementary file 1 — Data S1. [file FSN3-13-e70366-s001.docx]

SUPPLEMENTAL TABLE 1. Three-way Analysis of Variance (ANOVA) results determining the effect of storage time, wine replicate, and panelist on Chardonnay aroma attribute intensities.

| Aroma attribute | Source | DF | Sum of squares | Mean squares | F | Pr > F |
| --- | --- | --- | --- | --- | --- | --- |
| *Apple/Pear* | Model | 21.000 | 38.259 | 1.822 | 3.851 | **<0.0001** |
|  | Error | 140.000 | 66.235 | 0.473 |  |  |
|  | Corrected Total | 161.000 | 104.494 |  |  |  |
|  | Source | DF | Sum of squares | Mean squares | F | Pr > F |
| *Vegetal* | Model | 21.000 | 73.448 | 3.498 | 3.965 | **<0.0001** |
|  | Error | 140.000 | 123.496 | 0.882 |  |  |
|  | Corrected Total | 161.000 | 196.944 |  |  |  |
|  | Source | DF | Sum of squares | Mean squares | F | Pr > F |
| *Honey* | Model | 21.000 | 12.194 | 0.581 | 1.694 | **0.038** |
|  | Error | 140.000 | 47.985 | 0.343 |  |  |
|  | Corrected Total | 161.000 | 60.179 |  |  |  |
|  | Source | DF | Sum of squares | Mean squares | F | Pr > F |
| *Peach* | Model | 21.000 | 26.729 | 1.273 | 2.713 | **0.000** |
|  | Error | 140.000 | 65.672 | 0.469 |  |  |
|  | Corrected Total | 161.000 | 92.401 |  |  |  |
|  | Source | DF | Sum of squares | Mean squares | F | Pr > F |
| *Baking Spices* | Model | 21.000 | 0.937 | 0.045 | 1.599 | 0.057 |
|  | Error | 140.000 | 3.908 | 0.028 |  |  |
|  | Corrected Total | 161.000 | 4.846 |  |  |  |
|  | Source | DF | Sum of squares | Mean squares | F | Pr > F |
| *Vinegar/Nail Polish Remover* | Model | 21.000 | 73.637 | 3.507 | 6.180 | **<0.0001** |
|  | Error | 140.000 | 79.431 | 0.567 |  |  |
|  | Corrected Total | 161.000 | 153.068 |  |  |  |
|  | Source | DF | Sum of squares | Mean squares | F | Pr > F |
| *Banana* | Model | 21.000 | 25.270 | 1.203 | 2.418 | **0.001** |
|  | Error | 140.000 | 69.675 | 0.498 |  |  |
|  | Corrected Total | 161.000 | 94.944 |  |  |  |
|  | Source | DF | Sum of squares | Mean squares | F | Pr > F |
| *Butter* | Model | 21.000 | 2.102 | 0.100 | 1.381 | 0.138 |
|  | Error | 140.000 | 10.151 | 0.073 |  |  |
|  | Corrected Total | 161.000 | 12.253 |  |  |  |

SUPPLEMENTAL TABLE 2. Multivariate Analysis of Variance (MANOVA) results using Wilks’ Lamba Test for the effect of storage time, wine replicate, and panelist on Chardonnay aroma attribute intensities.

|  | Time (weeks) | Replicate | Panelist |
| --- | --- | --- | --- |
| Lambda | 0.375 | 0.969 | 0.092 |
| F Observed values | 2.664 | 0.464 | 2.976 |
| DF1 | 54 | 9 | 126 |
| DF2 | 678 | 132 | 1023 |
| F Critical value | 1.356 | 1.951 | 1.233 |
| *p*-value | <0.0001 | 0.896 | <0.0001 |

SUPPLEMENTAL TABLE 3. Model’s fit including *p*-value, R-squared, and F values for significant relationships displayed in TABLE 5.

| Variables | *p*-value | R-squared | F value |
| --- | --- | --- | --- |
| Honey | <0.0001 | 0.4948 | 18.61 |
| Sensor AHS | |  |  |
| Vinegar/nail polish remover | <0.0001 | 0.4895 | 18.22 |
| Sensor AHS | |  |  |
| Honey | 0.019 | 0.2573 | 6.58 |
| Sensor PKS | |  |  |
| Vinegar/nail polish remover | 0.018 | 0.2601 | 6.68 |
| Sensor PKS | |  |  |
| Vegetal | 0.019 | 0.2568 | 6.57 |
| Sensor CTS | |  |  |
| Honey | <0.0001 | 0.6181 | 30.75 |
| Sensor CTS | |  |  |
| Vinegar/nail polish remover | <0.0001 | 0.6017 | 28.7 |
| Sensor CTS | |  |  |
| Honey | 0.001 | 0.4785 | 17.43 |
| Sensor NMS | |  |  |
| Vinegar/nail polish remover | 0.014 | 0.279 | 7.35 |
| Sensor NMS | |  |  |
| Vegetal | 0.02 | 0.2545 | 6.49 |
| Sensor CPS | |  |  |
| Honey | <0.0001 | 0.4923 | 18.42 |
| Sensor CPS | |  |  |
| Vinegar/nail polish remover | <0.0001 | 0.601 | 28.61 |
| Sensor CPS | |  |  |
| Vegetal | 0.015 | 0.2715 | 7.08 |
| Sensor ANS | |  |  |
| Honey | 0.001 | 0.4576 | 16.03 |
| Sensor ANS | |  |  |
| Vinegar/nail polish remover | <0.0001 | 0.6255 | 31.73 |
| Sensor ANS | |  |  |
| Honey | 0.001 | 0.4697 | 16.83 |
| Sensor SCS | |  |  |
| Vinegar/nail polish remover | <0.0001 | 0.4901 | 18.27 |
| Sensor SCS | |  |  |
